# Succinic Acid Production With *Actinobacillus succinogenes* –Influence of an Electric Potential on the Intercellular NADH/NAD^+^ Balance

**DOI:** 10.1002/elsc.202400053

**Published:** 2024-11-13

**Authors:** Jan‐Niklas Hengsbach, Marcel Cwienczek, Wolfgang Laudensack, Judith Stiefelmaier, Nils Tippkötter, Roland Ulber

**Affiliations:** ^1^ Mechanical and Process Engineering RPTU Kaiserslautern‐Landau Kaiserslautern Germany; ^2^ Bioprocess Engineering and Downstream Processing University of Applied Science Aachen Jülich Germany

**Keywords:** *Actinobacillus succinogenes*, bioelectrochemical system, electrobiotechnology, NADH/NAD^+^, succinic acid

## Abstract

Bioelectrochemical systems (BESs) offer a sustainable method for chemical production, including the enhanced production of succinic acid. By combining fermentation with BES, it could be possible to achieve sustainable succinic acid production and CO_2_ fixation using *Actinobacillus succinogenes*. In literature, the potential application of BES is commonly associated with increased succinate yields, as it is expected to enhance the availability of NADH, thereby influencing the intracellular nicotinamide adenine dinucleotide (NADH/NAD^+^) balance. However, it remains unclear whether BES can improve NADH regeneration and achieve higher NADH/NAD^+^ ratios across all growth phases of *A. succinogenes*. This study investigates the impact of an applied electrical potential on the intracellular NADH/NAD^+^ ratio during an electrochemical‐assisted fermentation process. Using an adapted high‐performance liquid chromatography method with a Supelcosil LC‐18‐T column, it was demonstrated that NADH availability in BES, particularly during the stationary growth phase, improved by up to 1.98‐fold compared to the control. This enhancement in reducing power led to a succinate yield of 0.747 ± 0.01 g g^−1^, representing a 15.65% increase compared to a fermentation without electrochemical assistance. These findings support the expectation that the use of BES could enhance the competitiveness of bio‐based succinate production.

**Abbreviations**
BES: bioelectrochemical system
NADH/NAD^+^
: nicotinamide adenine dinucleotide
TCA
: tricarboxylic acid

## 1 Introduction

Energy conversion with electrochemical‐assisted fermentation (electro‐fermentations) using bioelectrochemical systems (BESs) has drawn increasing attention as the technology provides an alternative for sustainable chemical production. In electro‐fermentation, unlike microbial electrosynthesis, electrons are not the driving force of fermentation. Instead, they act as a trigger that influences the metabolic pathway by affecting the intracellular oxidation–reduction potential during self‐driven fermentations on organic carbon compounds [1]. One promising application of this technology is the enhanced production of succinic acid.

**Summary**

• The key objective of fermentation research is the cost‐efficient production of bioproducts, making it crucial to continuously develop and optimize cultivation methods to achieve high concentrations, productivity, and yields of desired products.

• Biobased succinic acid is a promising platform chemical with various applications, but it competes with petrochemical alternatives. A promising strategy to enhance the competitiveness of biobased products is the fermentation in bioelectrochemical systems (BESs).

• This study investigates the impact of an electric potential in a BES on the nicotinamide adenine dinucleotide (NADH/NAD^+^) ratio of *Actinobacillus succinogenes* and the associated succinate yield during electrochemical‐assisted fermentation.

• Our results show that NADH availability, especially during the stationary growth phase, can be improved, leading to increased reducing power and better succinate yield. We assume these findings could enhance the competitiveness of biobased succinic acid.

Succinic acid can be produced either from fossil fuels via petrochemical synthesis through the hydration of maleic acid or via microbial fermentation [2]. Microbial production involves using various organisms, such as fungi or bacteria, with the primary advantage being the fixation of carbon dioxide during fermentation [2, 3]. Another advantage is the independence from fossil fuels and the moderate reaction conditions. However, microbial fermentations face challenges such as the need for large capacities and the high dilution of the product. Therefore, to compete with the petrochemical process in terms of yield, cost, and production time, further optimization of fermentation processes is required [4]. By combining fermentation with a BES, succinic acid can be produced sustainably while simultaneously fixing CO_2_ [3]. Additionally, this approach supports Power‐to‐X strategies by converting renewable energy into high‐value chemicals, thereby promoting greener and more sustainable industrial processes.

BES offers a possibility to enhance fermentation and achieve higher yields by applying an electric potential to provide additional reduction equivalents to microorganisms [5]. The aim is to shift the metabolic pathway toward succinic acid production. Several studies have investigated the influence of an electric potential on succinic acid production with *Actinobacillus succinogenes* and *Escherichia coli* [6, 7, 8, 9, 10]. For *A. succinogenes*, succinic acid is a product of anaerobic metabolism, that is, highly dependent on the carbon dioxide concentration. The metabolic process involves two main pathways: the reductive branch of the tricarboxylic acid (TCA) cycle, which produces succinate, and the C3 pathway, which primarily results in formate and acetate. High concentrations of CO_2_ promote succinic acid production, whereas low concentrations lead to the formation of by‐products [11]. *A. succinogenes* produces succinate via the reductive branch of the TCA cycle, consuming 4 moles of NADH per 2 moles of succinate, while glycolysis only generates 2 moles of NADH. This redox cofactor imbalance leads to a shift toward C3 pathway for NADH regeneration [12]. The application of an electrical potential in BES is expected to increase NADH availability and influence the intracellular oxidation–reduction potential [1, 13]. However, for *A. succinogenes*, the effect on the nicotinamide adenine dinucleotide (NADH/NAD^+^) ratio in an electro‐biotechnological context has only been studied at a single time point, leaving it unclear whether BES improves NADH regeneration and increases NADH/NAD^+^ ratios throughout all growth phases of fermentation [14].

In this communication, we investigated the influence of an electric potential on the fermentation process of *A succinogenes*, focusing on its effect on succinate yield and the intracellular NADH/NAD^+^ ratio across all growth phases. A simple single‐chamber BES based on a 100‐mL Schott bottle with a typical three‐electrode setup was used. Additionally, we adapted a high‐performance liquid chromatography (HPLC) method for analyzing NAD^+^ and NADH in *A. succinogenes* samples. The resulting educt and product yields, as well as NAD^+^ and NADH concentrations, should provide insights into the benefits of using BES for biobased succinate production.

## 2 Materials and Methods

### 2.1 Microorganisms, Media, and Culture Conditions

The used strain in this study was *A. succinogenes* DSM 22257, strain 130Z (Leibniz Institute DSMZ German Collection of Microorganisms and Cell Cultures, Braunschweig, Germany). For all main and precultures, the corresponding medium described by Wang et al. was used [13] (see Supporting Information for details). Precultures were grown in 100 mL serum bottles with 50 mL of medium under carbon dioxide atmosphere. All serum bottles were inoculated with a 1 mL cryo culture and incubated for 24 h. Cultivations were performed at 37°C and 200 rpm.

### 2.2 Bioelectrochemical Setup

Main cultures of 100 mL were cultivated in a one‐chamber BES with a three‐electrode setup. Activated carbon felt ACC‐5092‐15 (KYNOL EUROPA GmbH, Hamburg, Germany) was used as a working electrode with a geometric surface area of 17.5 cm^2^. A graphite rod 9 cm in length with a diameter of 6 mm (Schuchardt Lehrmittel, Göttingen, Germany) was utilized as a counter electrode. An Ag/AgCl electrode (saturated KCl) (Sensortechnik Meinsberg, Waldheim, Germany) was used as a reference electrode. Additionally, neutral red was added to the main experiment and to the control in a concentration of 0.1 mmol L^−1^. During the fermentation, the pH of all BES was set to 6.8, and they were gassed with 0.2 vvm carbon dioxide. The applied potential of −800 mV was controlled using the multi‐channel potentiostat MultiEmStat3 (PalmSens, Utrecht, Netherlands). The descriptions of the statistics and equations used can be found in the Supporting Information.

### 2.3 Analytics

NADH and NAD^+^ were analyzed using a Waters HPLC system using the “Empower 3” software (Waters Corporation, Milford, USA). A SUPELCOSIL LC‐18‐T column (15 cm × 4.6 mm, 3 µm) and a Supelguard LC‐18‐T precolumn (2 cm × 4 mm, 5 µm) were used (Merck KGaA, Darmstadt, Germany). The column temperature was maintained at 25°C and the flow rate was set to 1 mL min^−1^. Eluents used were 0.05 M potassium phosphate buffer (pH 7) (Eluent A) and methanol (Eluent B). The gradient started with 100% Eluent A for 5 min, followed by an increase of B to 5% over 1 min, held for 5 min. Eluent B was then increased to 15% over 2 min and held for 10 min, afterward decreased to 0% over 1 min. At the end Eluent A was held at 100% for 6 min. A photodiode array detector (PDA 2996, Waters Corporation, Milford, USA) recorded a range from 190 to 400 nm, the evaluation was performed mainly at 261 nm, and if necessary, additionally at 340 nm. The injection volume was 100 µL. Detailed information for the extraction method can be found in the Supporting Information. The method for substrate‐product analysis used in this study has been previously described in detail and is well suited for the metabolites of *A. succinogenes* [15].

## 3 Results and Discussion

### 3.1 Electrochemical Assisted Fermentation Performance of *A. succinogenes*

Electro‐fermentation has emerged as a promising technique for enhancing microbial production. This section delves into the BES performance of *A. succinogenes* and how it can shift the metabolism toward increased succinate production by influencing intracellular redox potential.

**FIGURE 1:**
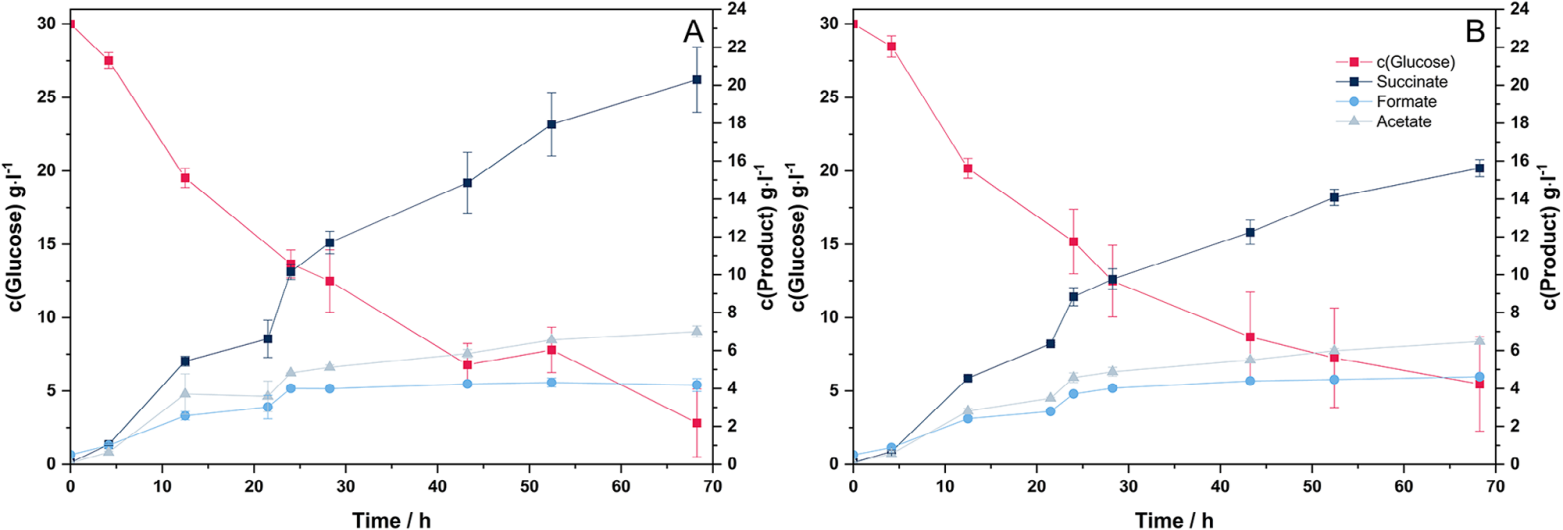
Educt and product concentrations of an electro‐fermentation of *Actinobacillus succinogenes* 130Z with 0.1 mmol L^−1^ neutral red, an applied potential of −800 mV (saturated KCl) (A) and the control experiment without potential (B). Fermentations were carried out with the following parameters: *T* = 37°C, *n* = 3, 200 rpm, pH 6.8 regulated with 5 M NaOH and gassing with 0.2 vvm CO_2_.

Figure 1 shows the results of a cathodic electro‐fermentation of *A. succinogenes* with a negative potential of −800 mV. The two diagrams illustrate the typical product distribution into succinic acid, formic acid, and acetic acid that occurs with increased CO_2_ availability. The final concentrations of the target product are 20.29 ± 1.17 g L^−1^ for the reactors with an applied potential and 15.62 ± 0.45 g L^−1^ in the control batch. In relation to the metabolized glucose, this results in succinic acid yields of 0.747 ± 0.01 and 0.646 ± 0.066 g g^−1^. In percentage terms, this corresponds to an increase in yield of 15.65% compared to the control. The final concentrations of the by‐products formic acid and acetic acid are 4.18 ± 0.34 and 7.00 ± 0.29 g L^−1^ with applied potential. In the control, the final concentration of formic acid is 4.61 ± 0.01 g L^−1^ and that of acetic acid is 6.47 ± 0.10 g L^−1^. Considering the normalized titers and the assumption that 4 mol of electrons are required in metabolism, a coulombic efficiency of 50.26% for the overproduction of succinate in the BES can be calculated theoretically in combination with the measured current response. Additionally, the carbon balance shows that in the control 56.05% ± 4.95% of the carbon atoms are bound in the end product succinate. In the BES, this value can be increased to 63.50% ± 0.11%. At the same time, the carbon content bound in the by‐products decreases by 3.44% compared to the control.

The results demonstrate a significant enhancement in succinate yield attributable to the applied electric potential, aligning well with previously reported results in the literature [7, 14, 16]. The observed increase in succinate yield in this study seamlessly corresponds to the previously described enhancements ranging from 7% to 36%. The high increases in concentration were achieved exclusively in two‐chamber systems [7, 8, 9, 14].

In the case of Pateraki et al. [7], which is the only fermentation described in the literature in a single‐chamber system, the increase in yield was 7%. This means that with our single‐chamber setup, we were able to increase the yield twice as high as previously described. Most likely, the mediator neutral red used is a significant factor here (Figure S1). For *A. succinogenes*, mediated electron transfer using mediators such as neutral red is described in the literature and at higher electric potentials hydrogen is discussed as a potential possibility [8, 13, 16]. Whether *A. succinogenes* is capable of direct electron transfer has not yet been demonstrated in practical experiments, which would explain the improved performance of the system in this study.

### 3.2 Analysis of NADH/NAD^+^ Ratios of *A. succinogenes* During Process Duration

Through succinate production via the reductive branch of the TCA cycle, *A. succinogenes* consumes 4 mol of NADH to produce 2 mol of succinate from 1 mol of glucose. However, only 2 mol of NADH is generated from 1 mol of glucose during the preceding glycolysis [12]. This results in a clear imbalance of redox cofactors, leading to a lack of reducing power in the metabolism. In the literature, the application of a potential in BES is commonly attributed to an increased yield of succinate, as it is expected to enhance NADH availability, which in turn should influence the intracellular oxidation–reduction potential [1, 10, 13]. Only Zhao et al. have investigated the assumed imbalance of the NADH/NAD^+^ ratio in the metabolism of *A. succinogenes* in an electro‐biotechnological context and found an apparently favored ratio toward NADH based on a single measurement time point [14]. However, whether the application of BES leads to improve NADH regeneration of *A. succinogenes* and thus to higher NADH/NAD^+^ ratios in all growth phases of the organism is not known over a complete fermentation run.

**FIGURE 2:**
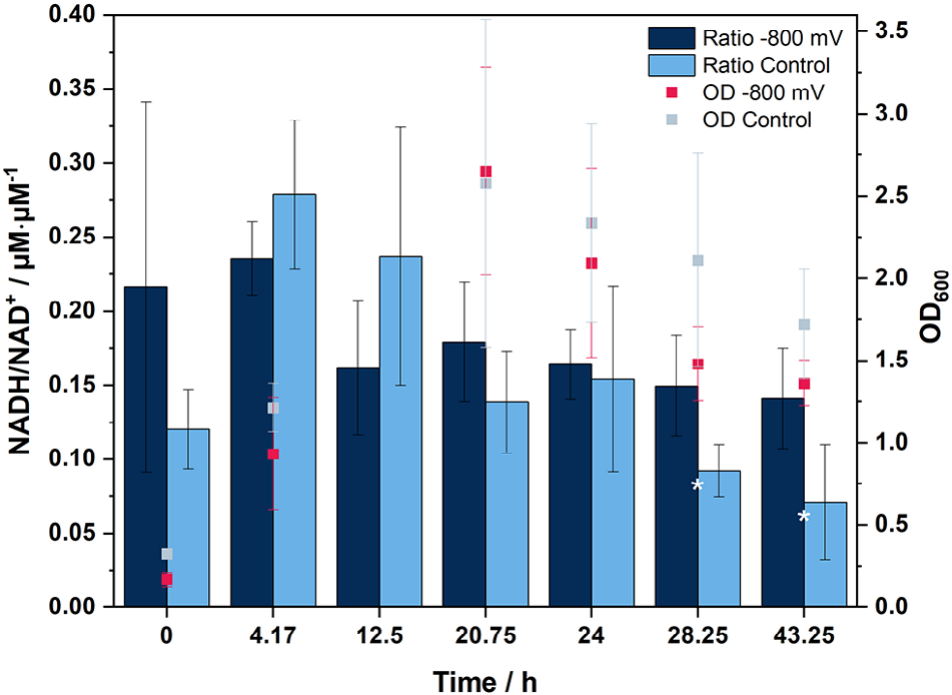
Comparison of electro‐fermentation and non‐electrical control fermentation regarding nicotinamide adenine dinucleotide (NADH/NAD^+^) ratio and optical density (OD_600_). Experimental parameters: *Actinobacillus succinogenes* 130Z in main culture medium with 0.1 mM neutral red, applied potential of −800 mV (saturated KCl), *T* = 37°C, 200 rpm, pH 6.8 regulated with 5 M NaOH and gassing with 0.2 vvm CO_2_. Each value is the average of three parallel biological replicates and is presented as mean ± standard deviation (*p < 0.1).

To further investigate this issue, an analytical method for the investigation of intracellular NAD and NADH was optimized for the production strain used. This method was applied to samples collected at different time points during an electro‐fermentation at −800 mV. The results show the progression of the NADH/NAD^+^ ratio over time (Figure 2). The highest ratio was measured after 4.17 h in both experiments, being 0.24 ± 0.02 in the electro‐fermentation and 0.28 ± 0.05 in the control. The ratio decreased over time, reaching 0.14 ± 0.03 in the electro‐fermentation and 0.07 ± 0.03 in the control after 43.25 h. Additionally, the OD_600_ values at the time of measurement are provided, showing a typical trend of anaerobic *A. succinogenes* fermentation, with a decrease in OD during the stationary phase [8, 14].

In addition to the decrease in OD after the exponential phase, a clear decrease in the NADH/NAD^+^ ratio can also be observed during this period. The organism therefore has decreasing amounts of NADH available over time during anaerobic cultivation. When these results are compared with studies on the influence of carbon sources with different oxidation states on the availability of NADH in the metabolism of *A. succinogenes*, a similar decreasing trend in NADH availability is evident. Using glucose as a carbon source, the NADH/NAD^+^ ratio decreases over time from 1.86 ± 0.32 to 0.49 ± 0.04 (Table 1) [17]. This indicates a limitation of NADH over time due to glucose as a carbon source, a phenomenon also observed in our BES. The larger discrepancy with the NADH ratios compared to the literature source is likely attributable to differences in sample preparation and analysis methods. Nevertheless, the results clearly demonstrate a positive effect of the applied electrical potential on fermentation, particularly evident for the stationary phase. Although, as described above, the general availability of NADH decreases over the course of the fermentation, the NADH levels are significantly higher in the electro‐fermentation compared to the control during the stationary phase. For instance, at the last two measurement points, there were significant improvements by factors of 1.62‐ and 1.98‐fold. This positive effect on the NADH/NAD^+^ ratio is therefore particularly noticeable after the exponential phase. No significant improvements in the ratio were observed before or during the exponential phase in this study. A deeper look into the literature confirms these findings. Thus, a fermentation study of *E. coli* for anaerobic succinate production demonstrates that controlling the oxidation–reduction potential at −400 mV results in an increased NADH/NAD^+^ ratio compared to −200 mV. The data were collected from a sample after 120 h of anaerobic fermentation, which, according to the indicated dry biomass, must have been at the end of the stationary phase [10]. For *A. succinogenes*, the measurement time for the NADH/NAD^+^ ratio reported by Zhao et al. is 24 h, corresponding to the early stationary phase based on the shown growth curve [14]. Therefore, all these factors suggest that applying an electrical potential to an *A. succinogenes* fermentation, particularly during the stationary phase, positively affects NADH availability.

**TABLE 1:** Comparison of succinate yields and titers with NADH/NAD^+^ ratios.

Organism	SA titer (g L^−1^)	SA yield (g g^−1^)	NADH/NAD^+^	Method	Reference
*E. coli* LL016	28.6^a^	0.76^b^, ^c^	0.11 − 0.20^d^	Enzyme cycling assay	[10]
*A. succinogenes* NJ113	26.4 ± 0.67^a^	0.66 ± 0.02	0.48 − 1.86^d^	Enzyme cycling assay	[17]
*A. succinogenes* NJ113	7.88 ± 0.24^a^	0.53 ± 0.07^b^, ^c^	0.34 − 0.53^d^	Enzyme cycling assay	[14]
*A. succinogenes* 130Z	20.29 ± 1.17	0.75 ± 0.01	0.24 − 0.28	HPLC	This study

Abbreviations: HPLC, high‐performance liquid chromatography; NADH/NAD^+^, nicotinamide adenine dinucleotide; SA, succinic acid.

^a^ Highest value from the corresponding reference.

^b^ Calculated according to values from the corresponding reference.

^c^ Approximate data taken from the diagram.

^d^ From several values from the corresponding reference.

## 4 Conclusion

In conclusion, this study investigated the effect of an electrical potential in a BES on the NADH/NAD^+^ ratio of *A. succinogenes* and the associated succinate yield over the duration of fermentation. It was demonstrated that NADH availability in a BES, especially during the stationary growth phase, could be improved by up to a factor of 1.98‐fold compared to the non‐electrical control. The enhanced reducing power in the organism resulted in a succinate yield of 0.747 ± 0.01 g g^−1^, corresponding to an increase of 15.65% compared to the control experiment. These findings support the expectation that the use of a BES for succinate production could increase its competitiveness against the petrochemical process.

## Conflicts of Interest

The authors declare no conflicts of interest.

## Supporting information



Supporting Information

## Data Availability

The data that support the findings of this study are available from the corresponding author upon reasonable request.
