# Supplementary material for: Succinic Acid Production With Actinobacillus succinogenes –Influence of an Electric Potential on the Intercellular NADH/NAD+ Balance
Source: Eng Life Sci. 2024 Nov 13;25(1):e202400053. doi: 10.1002/elsc.202400053 (PMC11717146; doi:10.1002/elsc.202400053)
Supplement: Supplementary file 1 — Supporting Information [file ELSC-25-e202400053-s001.pdf]

S1 Supporting information

S1.1 Extraction method

The extraction method was adapted from Hajjaj et al. [1]. A 75% ethanol solution with 10 mM HEPES buffer (pH 7.1) was brought to a boil. To the sample. 0.4 ml of the boiling solution was added. and afterwards incubated at 80°C and 600 rpm for 5 minutes. After cooling on ice for 10 minutes. the sample was frozen in liquid nitrogen and stored at -80°C. Before drying. the samples were centrifuged at 14000 rpm for one minute and then dried in a centrifuge vacuum concentrator at 43°C for 1.5 hours. The dried pellet was resuspended in 2 mL PBS buffer. centrifuged again and the supernatant filtered.

S1.2 Analysis of variance

Analysis of variance (ANOVA) was performed on molar $NADH/NAD^+$ ratios. The ratios of individual replicates were indexed as electro-fermentation or non-electric control fermentation. A one-way ANOVA was performed in the software OriginLab. Difference between means was considered significant, if the p-value was larger than 0.1.

S1.3 Cyclic voltammetry

A single-chamber system (BVT Technologies, Strázek, Czech Republic) was used to perform cyclic voltammetry (CV) measurements. The system consists of a TC 5 measuring cell with jacket cooling, a glass carbon electrode (WCEc.W4) as AE, a platinum electrode (ACEc.E1) as GE and an Ag/AgCl (saturated KCl) (RCEc.RS.E1) as RE. Before each measurement, the setup was cleaned electrochemically by performing 50 CV cycles in 0.5 M sulfuric acid over a range of -2 to 2 V at a scan rate of 0.5 $V \cdot s^{-1}$. For measurement, the electrolyte was filled into the chamber and CV was performed at room temperature. Scan rates ν and measuring ranges were varied. Potassium phosphate buffer and the Wang cathode medium were used as buffer for the mediator.

S1.4 Equations

S1.4.1 Carbon fixing/Recovery efficiency (%)

$$\eta(\%) = \frac{n_{succinate\ (\Delta t)} \times fC_{succinate}}{n_{glucose\ (\Delta t)} \times fC_{glucose} + n_{\mathrm{CO2}\ (\Delta t)}} \times 100 \quad (2)$$

$\eta(\%)$	Carbon fixing/Recovery efficiency	$n_{\mathrm{glucose}\ (\Delta t)}$	Amount of glucose (mol) at (t_0-t_1)
$n_{\mathrm{succinate}\ (\Delta t)}$	Amount of succinate (mol) at (t_1-t_0)	$fC_{succinate}$	Moles of carbon in one mole of succinate
$fC_{glucose}$	Moles of carbon in one mole of glucose	$n_{\mathrm{CO2}\ (\Delta t)}$	Amount of CO_2 bound in succinate<br>1 mol succinate = 1 mol CO_2

S1.4.2 Yield

$$Y_{P/S} = \frac{(P_{t1} - P_{to})}{(S_{t0} - S_{t1})}$$

(3)

$Y_{P/S}$	Yield (g g^{-1})	P_{to}	Amount of product at t_0
P_{t1}	Amount of product at t_1	S_{t0}	Amount of substrate at t_0
S_{t1}	Amount of substrate at t_1		

S1.4.3 Coulombic efficiency (CE%)

$$CE(\%) = \frac{nFm(\Delta succinate)}{\int_0^t I\,dt} \times 100$$

(3)

$CE(\%)$	Coulombic efficiency (%)	m	Amount of succinate (mol) for $(\Delta succinate_{BES} - \Delta succinate_{control})$
n	Electrons (mol) required in metabolism	$\int_0^t I\,dt$	Integral of current response = number of electrons transferred
F	Faraday constant		

S1.5 Composition of growth media

S1.5.1 Composition of pre-culture [2]

Components	Concentration [g·L^{-1}]
NaCl	1.0
$NaHCO_3$	10.0
$NaH_2PO_4 \cdot 2xH_20$	9.6
$K_2HPO_4 \cdot 3xH_20$	15.5
Corn steep liquor	2.5
Yeast extract	5.0
D(+)-Glucose	5.0

S1.5.2 Composition of Wang cathode media [2]

**Components**	**Concentration [g·L^{-1}]**
$NaHCO_3$	10.0
$Na_2HPO_4 \cdot 12\ H_2O$	31.5
$NaH_2PO_4 \cdot 2\ H_2O$	8.5
Yeast extract	5.0
D(+)-Glucose	30

S1.6 Cyclic voltammograms of neural red

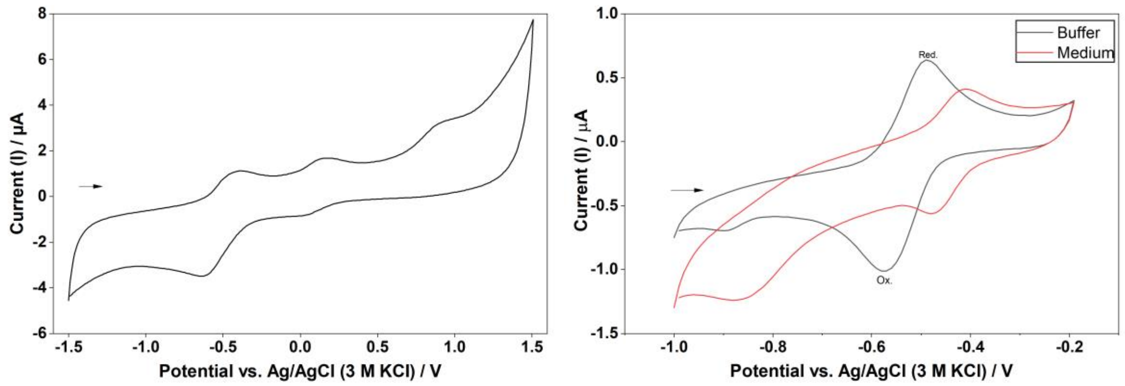

Figure S1: Different cyclic voltammograms of neural red in different buffer concentrations and the main culture medium. Left: 5 mmol·l^{-1} neutral red in 100 mM potassium phosphate buffer ($\nu = 0.2$ V·s^{-1}). Right: 0.1 mmol·l^{-1} neutral red in 1 M potassium phosphate buffer in gray or main culture medium in red ($\nu = 0.5$ V·s^{-1}). The arrow indicates the direction of measurement. Experimental parameters: Test parameters: T = 24 °C, pH 7, AE: glassy carbon, GE: platinum, RE: Ag/AgCl (KCl saturated).

References

[1] H. Hajjaj. P. . Blanc. G. Goma. and J. Francois. “Sampling techniques and comparative extraction procedures for quantitative determination of intra- and extracellular metabolites in filamentous fungi.” *FEMS Microbiol. Lett.*. vol. 164. no. 1. pp. 195–200. Jul. 1998

[2] Z. Wang *et al.*. “Enhanced succinic acid production from polyacrylamide-pretreated cane molasses in microbial electrolysis cells.” *J. Chem. Technol. Biotechnol.*. vol. 93. no. 3. 2018
